# *Ziziphus spina*-*christi* leaf extract ameliorates schistosomiasis liver granuloma, fibrosis, and oxidative stress through downregulation of fibrinogenic signaling in mice

**DOI:** 10.1371/journal.pone.0204923

**Published:** 2018-10-01

**Authors:** Rafa S. Almeer, Manal F. El-Khadragy, Semlali Abdelhabib, Ahmed E. Abdel Moneim

**Affiliations:** 1 Department of Zoology, College of Science, King Saud University, Riyadh, Saudi Arabia; 2 Department of Zoology and Entomology, Faculty of Science, Helwan University, Cairo, Egypt; 3 Genome Research Chair, Department of Biochemistry, College of Science, King Saud University, Riyadh, Saudi Arabia; Alexandria University, EGYPT

## Abstract

Schistosomiasis is a widespread parasitic infection that affects humans, as well as wild and domestic animals. It ranks second after malaria, with a significant health and socio-economic impact in the developing countries. The objective of this study was to assess the anti-schistosomal impact of *Ziziphus spina*-*christi* leaf extract (ZLE) on *Schistosoma mansoni*-induced liver fibrosis in CD-1 Swiss male albino mice. *S*. *mansoni* infection was achieved by dipping of mouse tails in schistosomal cercariae. ZLE treatment was initiated at 46 days post-infection by administering a dose of the extract on a daily basis for 10 consecutive days. *S*. *mansoni* infection resulted in liver granuloma and fibrosis, with a drastic elevation in liver function factors, nitric oxide, and lipid peroxidation, which were associated with a reduction in glutathione content and substantial inhibition of antioxidant enzyme activities compared to those of the control. Induction of hepatic granuloma, oxidative stress, and fibrosis in the liver was controlled by ZLE administration, which also produced inhibition of matrix metalloproteinase-9, alpha-smooth muscle actin, transforming growth factor-β, and tissue inhibitors of metalloproteinases expressions. In addition, the *S*. *mansoni-*infected group exhibited an increase in Bax and caspase-3 levels and a decrease in Bcl-2 level. However, treatment with ZLE mainly mitigated apoptosis in the liver. Thus, the findings of this study revealed that *Ziziphus spina*-*christi* had anti-apoptotic, anti-fibrotic, antioxidant, and protective effects on *S*. *mansoni-*induced liver wounds. The benefits of *Ziziphus spina*-*christi* extract on *S*. *mansoni* were partly partially mediated by enhancing anti-fibrinogenic and nuclear factor erythroid 2–related factor 2 (Nrf2) pathways.

## Introduction

Schistosomiasis, also known as bilharzia, is an infectious disease in humans and animals caused by trematode flatworms belonging to the genus *Schistosoma*. Schistosomiasis is classified as category II disease and ranks second after malaria as a significant tropical disease [1]. Previous studies have shown that more than 90% of the infected population from 74 developing countries was living in sub-Saharan Africa. An estimated 280,000 deaths in Africa are attributed to renal failure or bladder cancer. Moreover, a similar number of people in the Sahara are negatively impacted by portal hypertension, liver fibrosis, and schistosomiasis (caused by *Schistosoma haematobium* or *S*. *mansoni*). Approximately 240 million people are infected worldwide and approximately 800 million are reported to be at risk for infection [2]. Schistosomiasis has been effectively eliminated from Japan and Tunisia. Moreover, tremendous progress has been achieved in controlling schistosomiasis in Morocco and some of the Caribbean Islands countries. Additionally, stringent measures are being undertaken by China, Egypt, and Brazil for eliminating this disease [3].

An exaggerated increase in deposition of extracellular matrix (ECM) components has been reported in fibrosis, including category III collagen in fibrotic course, as well as type II along with type I collagen, fibronectin, and proteoglycan during several phases of granulomas development [4]. Activated hepatic stellate cells (HSCs) are the main source of extracellular matrix proteins in liver fibrogenesis [5]. Fibrogenic cytokines, such as transforming growth factor-β (TGF-β), belong to the main class of cytokines engaged in stimulation of fibrogenesis, which enabling enriched matrix synthesis and proliferation of HSCs [6]. Matrix metalloproteinases (MMPs) are the key enzymes that degrade different types of collagen. In liver, MMP-1 is synthesized in abundance by stimulated fibroblasts and HSCs [7]. Although, cirrhosis is irreversible, whereas fibrosis is reversible; therefore, it is vital to prevent progression of fibrosis to cirrhosis. However, an effective anti-fibrotic medication has not been identified to date. The existing treatments for several incurable liver sicknesses are impractical and ineffective, with liver transplantation as the only alternative; thus, there is an urgent need to develop innovative techniques to combat this problem [8].

Unfortunately, the therapeutic dose of the main anti-schistosomal drug, praziquantel (PZQ), does not markedly affect the damage produced by *S*. *mansoni* ova. PZO exhibit a limited effect once liver and spleen lesions have developed. Further, there is an increasing concern regarding the development of PZQ resistance [9], and this has encouraged the scientific community to develop innovative and low-cost treatment against schistosomiasis that could provide hepatic protection. Plant therapy has played an important role in this regard [1], and the search for antiparasitic compounds from natural sources, especially medicinal plants, has intensified [9].

*Ziziphus spina*-*christi* (L.) (ZSC) belongs to the Rhamnaceae family. It is a tropical evergreen tree that produces small, orange-yellow fruits. The wild plants are found in mainly in Sinai (in Egypt). It is commonly called Nabka and Sidr in other Middle Eastern countries [10]. Phytochemical evaluation has confirmed that ZSC contains essential oils, cyclopeptide alkaloids, tannins, phytosterols, flavonoids, triterpenoid sapogenins, and saponins, [11]. ZSC is utilized for its antimicrobial, hypoglycemic, immunostimulant, hepatoprotector, and tonic activities in traditional medicine [10, 12–13]. However, to the best of our knowledge, no studies have focused on the schistosomacidal activity of ZSC.

## Materials and methods

### Experimental animals

Group of 42 male mice (CD-1 Swiss albino) with weights ranging from 18 g to 22 g were purchased [from Schistosome Biology Supply Center (SBSC)], Theodor Bilharz Research Institute (TBRI), Giza, Egypt. A standard commercial pelleted diet was provided to the mice and they were housed under standard laboratory conditions of light and temperature and under pathogen-free conditions. All protocols and animal handling procedures were approved by the Committee on Research Ethics for Laboratory Animal Care at the Department of Zoology, Faculty of Science, Helwan University (approval no, HU2017/Z/06).

### Preparation of *Ziziphus spina-christi* leaf extract

*Ziziphus spina-christi* leaves were purchased from the local market in Cairo, Egypt. Leaves were chosen and proven by an expert taxonomist from Botany Department, Faculty of Science, Helwan University, Egypt. *Ziziphus spina-christi* leaves were washed under running tap water, and then air-dried in shade. The dried ZSC leaves were finely ground and immersed in 70% (v/v) methanol at 4°C for 48 h. The extract was evaporated under reduced pressure to a semi-dry state utilizing a rotary evaporator at 45°C and then liquefied in distilled water. The extract thus obtained was designated as *Ziziphus spina-christi* leaf extract (ZLE) and stored at -20°C until further analysis.

### Determination of total phenols and flavonoids

Total polyphenols (TP) level was determined by the Folin–Ciocalteu method, and the total flavonoids (TF) level was determined using a AlCl_3_ method as previously described by Abdel Moneim [14]. Both TP and TF levels were expressed as milligram gallic acid equivalents per gram dried weight of the extract (mg GAE/g DW) and milligram quercetin equivalents per gram dried weight of the extract (mg QE/g DW) using the calibration curves of gallic acid and quercetin.

### Mice infection

*Schistosoma mansoni* cercariae were obtained from Schistosoma Biological Supply Center at TBRI. The experimental mice were exposed to *S*. *mansoni* (70 ± 5 cercariae/mouse) utilizing tail dipping process, as described previously [1].

### Design of experimental

Mice were divided into six groups of seven mice each. The first group (called Group I) was the normal control group, in which 100 μl of water was orally administered to each mouse for 10 days and the second group (Group II) was the ZLE control group, in which 400 mg/kg body weight (bwt) ZLE was orally administered daily for 10 days. Groups VI, V, IV, and III were infected with *S*. *mansoni*. Group III served as vehicle control, where starting from day 46 post-infection with *S*. *mansoni*, 100 μl of water was orally administered for 10 consecutive days to each mouse. A dose of 500 mg PZQ (dissolved in 70% glycerin) per kg bwt was orally administered to Group IV mice for 2 successive days. Mice in Groups V with VI were orally administered with 100 μl of 200 and 400 mg/kg between ZLE, respectively, every day and for 10 days. The treatments were started on day 46 post-infection (PI) to allow clinical expression of the disease and to evaluate the effectiveness of the extracts in alleviating the pathophysiological aspects of the sickness.

Twenty-four h after administration of the last dose on day 56 post infection with *S*. *mansoni*, the mice were euthanized with sodium pentobarbital (300 mg/kg bwt, Sigma-Aldrich), and then blood samples were taken for serum analysis. Livers were also dissected and rinsed in ice-cold 50 mM Tris–HCl, pH 7.4, twice. Livers were then individually weighed and directly homogenized in ice-cold 50 mM Tris–HCl, pH 7.4, to give a 10% (w/v) homogenate. The homogenates were centrifuged at 1000 *g* for 10 min at 4°C. The obtained supernatants were utilized for different biochemical analysis. The total protein concentration of the liver specimens was assayed by the Lowry’s technique [15] using bovine serum albumin (BSA) as a standard.

### Liver perfusion

Mice from each infected group were perfused to recover the adult *S*. *mansoni* worms using a modified method of Aly and Mantawy [16]. The worms from each mouse were allowed to settle for around 20 min in a 20-cm glass Petri dish, and then their number was determined and sex was specified. The protection degree (% decline in challenge) was measured as “Percentage worm reduction = C − T/C × 100.” C is defined as mean number of worms in untreated infected mice while T is the mean number of the worms in treated mice.

### Ova count

Egg burden was estimated by overnight incubation of liver tissue in 5% KOH at 37°C and then the recovered eggs were counted in 50 μl aliquots. The experiment was measured in triplicate and the ova count was presented as ova/g tissue.

### Test of function of liver

Serum aspartate aminotransferase (AST) and alanine aminotransferase (ALT) activity was determined colorimetrically by estimating the amount of oxaloacetate or pyruvate released as a result of the production of -2, -4-dinitrophenylhydrazine, as per the Reitman and Frankel method [17].

### Oxidant/antioxidant parameters

Lipid peroxidation (LPO) levels, expressed in terms of the amount of malondialdehyde (MDA) formed, were determined from the liver homogenates [18]. Nitrite/nitrate (nitric oxide; NO) and glutathione (GSH) were determined utilizing the protocols described by Green et al. [19] and Ellman [20], respectively. To assay the activities of antioxidant enzyme, superoxide dismutase (SOD) activity was measured through a technique illustrated by Nishikimi et al. [21], catalase was expressed by Aebi [22], glutathione peroxidase (GSH-Px) according to Paglia and Valentine [23], and glutathione reductase (GSH-R) as described by Factor et al. [24].

### Histopathological examination

Five mice in each group were randomly selected and various parts of the liver specimens were fixed in 10% phosphate buffered formalin, embedded in paraffin, sectioned at 5 μm thickness, and stained with hematoxylin and eosin, Sirius red, or Masson’s trichrome (for histopathological assessment combined with the analysis of the image). Unstained sections were employed for immunohistochemical analyses. Granuloma size (μm^2^) was measured in infected groups. The mean size was calculated from the average of width and length. At a minimum, 100 granulomas were examined in each group using ImageJ software (U.S. National Institutes of Health, Bethesda, MD, USA) and the size was expressed as the mean size ± standard deviation (SD).

### Immunohistochemical studies

Immunohistochemical studies of TIMP-2, MMP-9, TGF-β and caspases-3 were performed on 4- to 5-μm-thick sections. After deparaffinization and blocking the endogenous peroxidases, sections were incubated with mouse anti-TIMP-2, anti-MMP-9, anti-TGF-β or anti-caspases-3 (diluted 1:200, Santa Cruz Biotechnology, Santa Cruz, CA, USA) antibodies approximately 60 min at room temperature followed by the secondary antibody (dilution 1/2000, Thermo Fisher Scientific Inc., Waltham, MA, USA) and diaminobenzidin (DAB) to develop staining. Samples were stained with Mayer’s hematoxylin for 1 min and fixed utilizing Aquatex liquid (Merck KGaA, Germany). All samples were treated keeping similar conditions having similar antibody concentration to guarantee the immunostaining would be comparable across various experimental groups.

In the immunohistochemically-stained tissues, the color intensity for the immunoreactivity of each protein was semi-quantitatively evaluated in three randomly selected fields from five different specimens. The intensity was expressed as + (weak immunoreactivity), ++ (moderate immunoreactivity), +++ (strong immunoreactivity), or ++++ (very strong immunoreactivity).

### Molecular assay (real-time PCR)

Total RNA was separated from liver specimens utilizing a TRIzol-based method (Invitrogen, Carlsbad, CA, USA). One microgram total cellular RNA was reverse transcribed for the synthesis of first-strand cDNA utilizing Script cDNA synthesis kit (Bio-Rad, CA). For real-time PCR analysis, 10 ng cDNA was used for every PCR assay. All PCR assays were performed in triplicates utilizing Power SYBR Green (Life Technologies, CA, USA) on an Applied Biosystems 7500 Instrument. Gene expression was normalized to the expression of housekeeping gene GAPDH, and the relative gene expression values were estimated. The PCR primers (S1 Table) for the SOD2, CAT, GSH-Px1, GSH-R, Nrf2, IL-1β, TNF-α, Cox-2, αSMA, and TGF-β1 genes were synthesized by Jena Bioscience GmbH (Jena, Germany). Primers were designed using the Primer-Blast program from NCBI. The PCR primer sequences were BLAST searched to assure specificity for the particular gene.

### Statistical analysis

The data are expressed as mean ± SD. One-way ANOVA and post hoc Duncan’s test was used for comparisons between more than two groups utilizing the statistical package (SPSS version 17.0). p<0.05 was considered as significant for all statistical analyses.

### Results

Based on Folin-Ciocalteu and AlCl_3_ methods, the total polyphenols level was 87.3 mg GAE/g DW whereas, the flavonoids level was 9.6 mg QE/g DW (Table 1). These results were in consistent with the previous work of Elaloui et al. [25].

**Table 1:** Total polyphenols and flavonoids levels of *Ziziphus spina*-*christi* leaf extract.

Parameters	*Ziziphus spina*-*christi* leaf extract
Total polyphenols (mg GAE/g DW)	87.3±10.6
Total flavonoids (mg QE/g DW)	9.6±1.2

**Fig 1:**
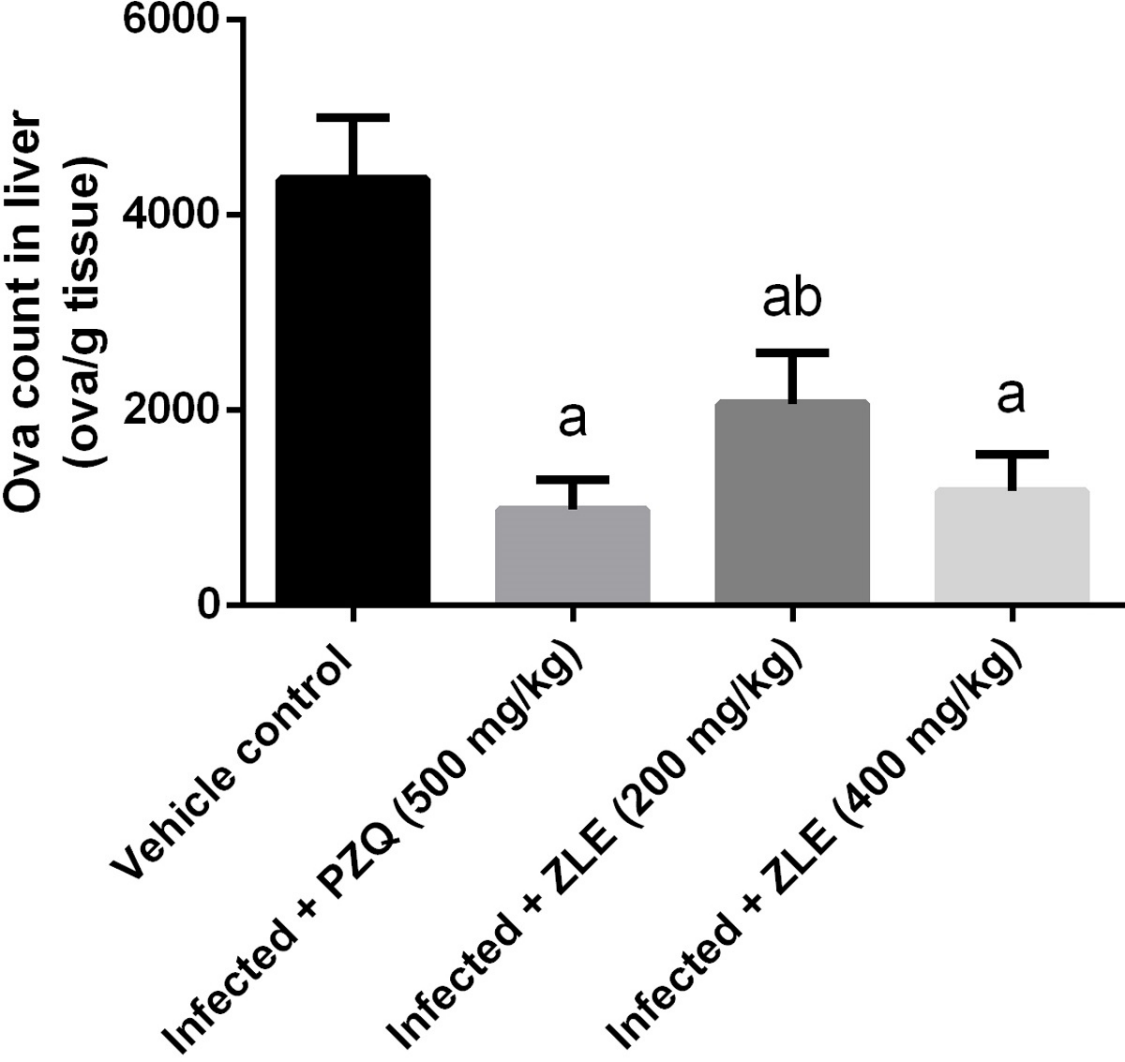
Ameliorative effects of the administration of *Ziziphus spina*-*christi* leaf extract (ZLE) on the ova count in the liver of CD-1 Swiss mice infected with *S*. *mansoni*. After 56 days, liver tissue was digested using 5% KOH and the recovered eggs was countted. Results (mean ± SD of triplicate measurements) ^a^*p*<0.05, significant change with respect to **Vehicle control group**; ^b^*p*<0.05, significant change with respect to **Infected+PZQ group** for Duncan's post hoc test.

The worm burden and ova count in infected and treated animals are presented in Table 2. Infected mice medicated with 200 or 400 mg/kg ZLE showed a significant drop (p<0.05) in the number of *S*. *mansoni* worms improved from infected mice by 51.1% and 64.2%, respectively. Also, ova count in these ZLE treated infected mice was decreased by 50% and 69.1%, respectively (p<0.05). Furthermore, the oogram pattern following ZLE administration commencing at day 46 post-infection revealed total fading of both immature and mature ova (data not shown). PZQ treatment at day 46 after the infection resulted in the death of all the ova, as anticipated. Moreover, data illustrated in Fig 1 revealed that a significant decline in the mean number of eggs/g liver tissue to 984 in PZQ (500 mg/kg), 1173 in ZLE (400 mg/kg) and 2068 in ZLE (200 mg/kg) compared to 4368 in the infected group.

**Table 2:** Effect of *Ziziphus spina-christi* leaf extract (ZLE) administration on mean worms number, percentage worm reduction, ova count and granuloma size in liver of *S*. *mansoni* infected mice.

Groups	Mean number of worms ± SEM	Percentage worm reduction (%)	Ova count in liver (ova/g tissue) ± SEM	Reduction on ova count (%)	Granuloma size (μm^2^)
Vehicle control	17.6 ±2.3	-	4368±627	-	1047862±1879
Infected+PZQ (500 mg/kg bwt)	5.2±0.7^a^	70.5^a^	984±308^a^	77.4^a^	921471±1473
Infected+ZLE (200 mg/kg bwt)	8.6±2.3^a^^b^	51.1^a^^b^	2068±526^a^^b^	52.6^a^^b^	856321±842^a^^b^
Infected+ZLE (400 mg/kg bwt)	6.3±0.7^a^^b^	64.2^a^	1173±376^a^	73.1^a^	745368±1065^a^^b^

Values are means ± SD (n = 7)

^a^*p*<0.05, significant change with respect to **Vehicle control group**;

^b^*p*<0.05, significant change with respect to **Infected+PZQ group** for Duncan's post hoc test.

**Fig 2:**
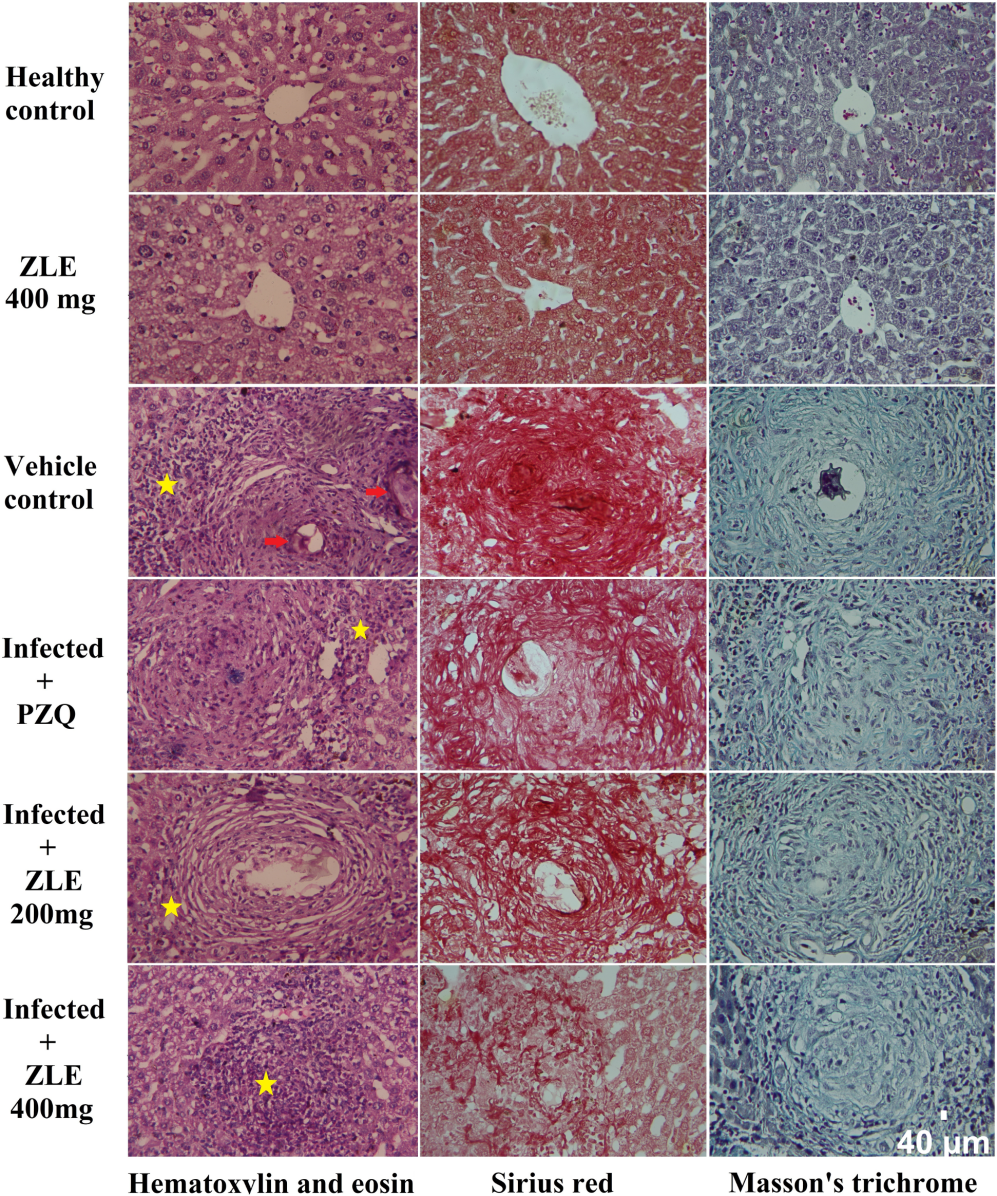
ZLE ameliorates liver histology of *S*. *mansoni* infected CD-1 Swiss mice stained with hematoxylin and eosin, Sirius red or Masson's trichrome (Original magnification 400×). Images representative liver histology of male CD-1 Swiss mice infected with 70±5 *S*. *mansoni* cercariae for 46 days receiving saline (healthy control), vehicle (infected mice received saline), PZQ (500 mg/kg PZQ for 2 successive days) and ZLE 200 and 400 mg/kg for 10 days. Yellow star indicate infiltrated leukocytes around granulomatous lesions and red arrow indicate schistosome eggs.

Histopathological examinations confirmed the parasitological findings (Fig 2). Liver tissue from mice infected with *S*. *mansoni* showed the presence of cellular and fibrous granuloma with necrosis at the center, condensed fibrous connective tissue, and centrally localized embedded ova with infiltrated leukocytes surrounding the granuloma. An obvious improvement was noticed in the ZLE-treated mice characterized by an attenuation in granuloma and fewer centrally localized ova with less condensed connective tissue. Collagen deposition and liver fibrosis were evaluated with Sirius red or Masson's trichrome, producing collagen fibers stained red or blue colors, respectively. In the current study, the amount of collagen fibers was increased markedly in *S*. *mansoni*-infected liver and collagen deposition was decreased gradually with ZFE treatments. Consistent with these results, the area of granulomas was alerted and the size decreased in ZLE-treated mice (Table 2). Interestingly, treatment with ZFE at 400 mg/kg caused a reduction in granuloma area that was similar to that with PZQ, a standard medicine used for treating schistosomiasis.

Levels of the serum transaminases, ALT and AST, were assessed to determine the degree of hepatocyte injury. As shown in Table 3, untreated infected mice showed a significant upregulation in the activity of the two enzymes, demonstrating that *S*. *mansoni* induces liver injury. An increase in these two liver functional parameters was also observed in the PZQ-administered mice. In contrast, the ZLE (200 and 400 mg/kg) treatment groups displayed attenuated liver injury, as indicated by a significant decrease in ALT and AST levels compared with those in the model mice.

**Table 3:** Serum ALT and AST of the studied groups.

Groups	ALT (IU/L)	AST (IU/L)
**Healthy control**	45.6±5.3	52.3±4.4
**ZLE** (400 mg/kg bwt)	42.1±3.6	49.3±3.7
**Vehicle control**	87.3±6.4^a^	92.7±6.4^a^
**Infected + PZQ** (500 mg/kg bwt)	79.2±5.8^a^	74.3±8.2^a^^b^
**Infected + ZLE** (200 mg/kg bwt)	68.7±5.2^a^^b^	68.6±4.1^a^^b^
**Infected + ZLE** (400 mg/kg bwt)	54.2±3.8^a^^b^	58.5±6.1^b^

All results are expressed as the mean ± SD (n = 7).

^a^p<0.05, significant change with respect to the **Control** group;

^b^p<0.05, significant change with respect to the **Infected** group using Duncan's post hoc test.

**Fig 3:**
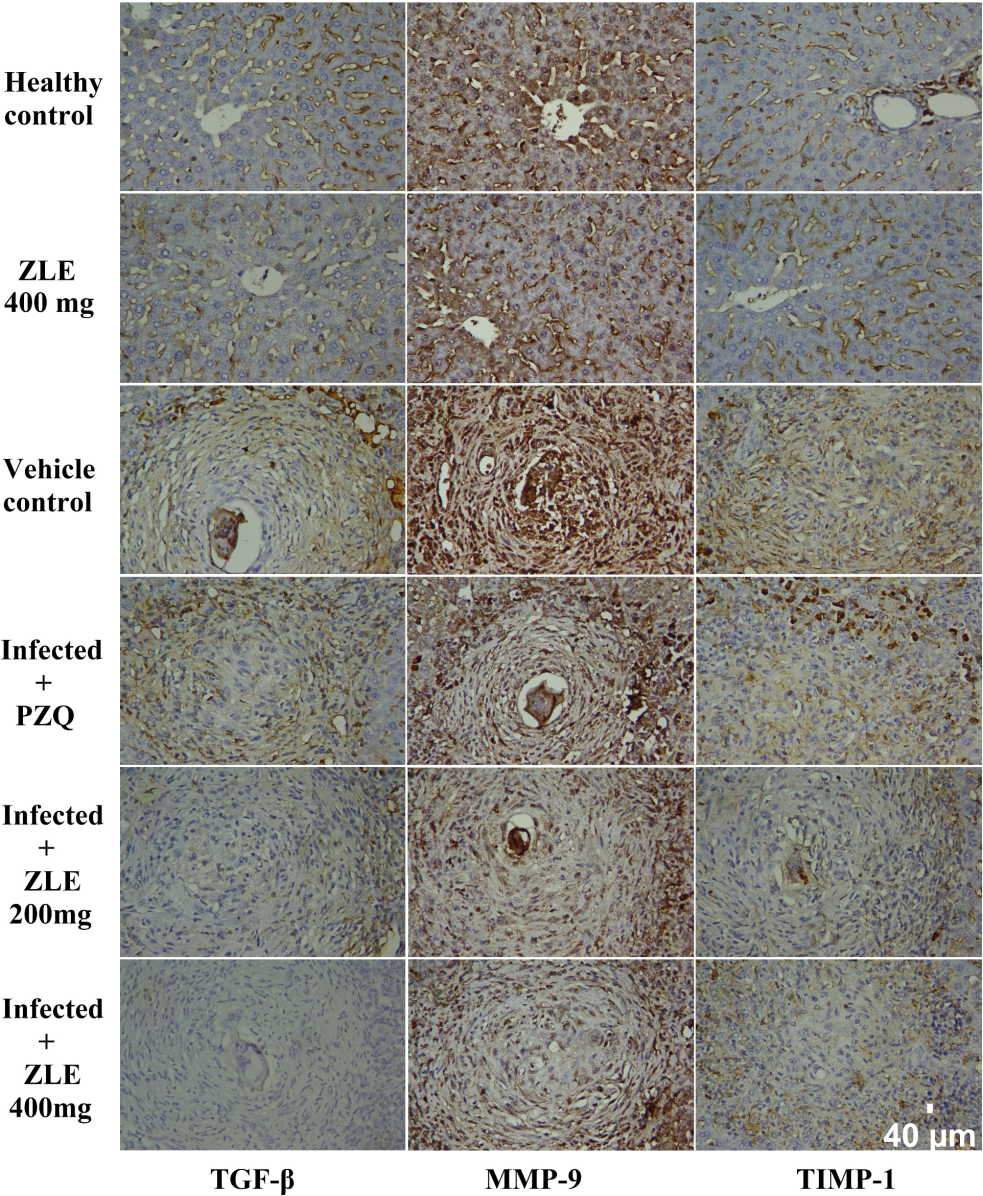
ZLE ameliorates liver fibrosis of *S*. *mansoni* infected CD-1 Swiss mice. Images representative immunohistochemistry for TGB-β, MMP-9 and TIMP-1 in hepatic tissue of male CD-1 Swiss mice infected with 70±5 *S*. *mansoni* cercariae for 46 days receiving saline (healthy control), vehicle (infected mice received saline), PZQ (500 mg/kg PZQ for 2 successive days) and ZLE 200 and 400 mg/kg for 10 days. (Original magnification 400×).

Immunohistochemical examinations of normal and untreated infected liver tissues showed negative or low immunoreactivities for MMP-9, TIMP-1, and TGF-β in hepatic tissue (Fig 3). In the PZQ-treated group, moderate TIMP-1 and strong MMP-9 and TGF-β immunoreactivities were observed in liver tissues adjacent to granulomas. ZLE administration resulted in only mild TIMP-1, MMP-9, and TGF-β immunoreactivities neighboring granulomas in liver tissues, showing that ZLE has an anti-fibrotic action. Compared with the infected mice, ZFE (400 mg/kg) treatment showed marked alternation in these markers (S2 Table).

**Fig 4:**
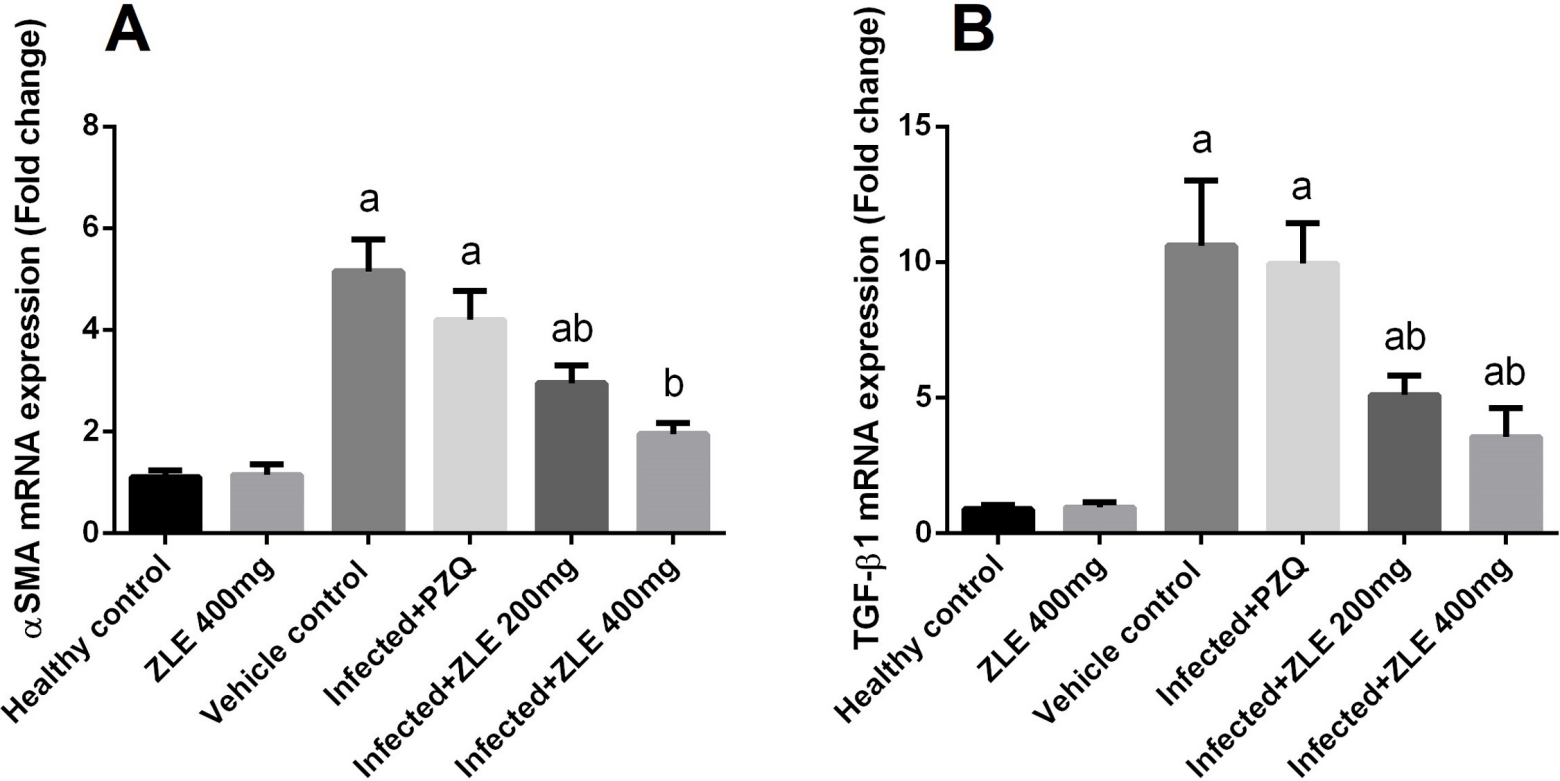
Ameliorative effects of the administration of *Ziziphus spina*-*christi* leaf extract (ZLE) on the mRNA levels of α-SMA and TGF-β gene in the liver of CD-1 Swiss mice infected with *S*. *mansoni*. After 56 days, liver homogenates were prepared to assayed α-SMA (A) and TGF-β (B) genes expression using RT-PCR method. Results (mean ± SD of three independent assays) were normalized to the GAPDH mRNA level and are shown as fold induction relative to the mRNA level in the control. ^a^p<0.05, significant change with respect to the **Control** group; ^b^p<0.05, significant change with respect to the **Infected** group using Duncan's post hoc test.

To determine whether ZLE has an anti-fibrotic activity, the expression of α-SMA and TGF-β was determined. Treatment with ZLE was sufficient in downregulating α-SMA and TGF-β expressions (Fig 4), whereas PZQ did not suppress all types of fibrosis-related genes in the liver.

**Fig 5:**
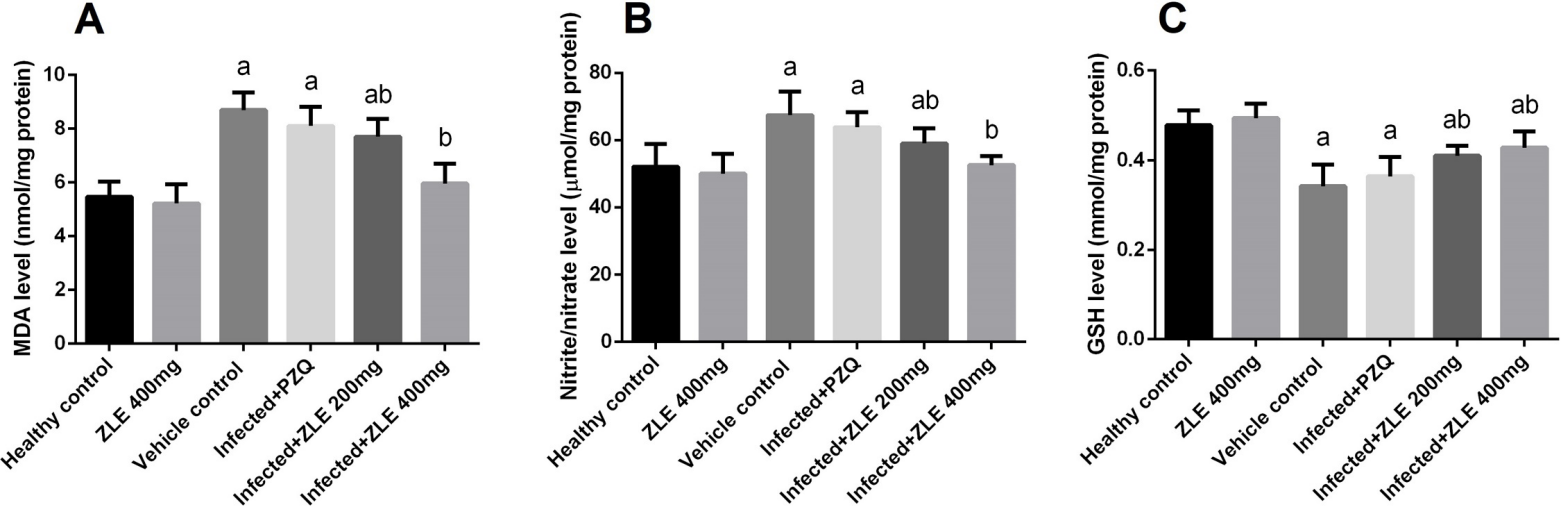
Ameliorative effects of the administration of *Ziziphus spina*-*christi* leaf extract (ZLE) on the oxidative stress markers levels in CD-1 Swiss mice infected with *S*. *mansoni*. After 56 days, liver homogenates were prepared to assayed LPO (A), NO (B) and GSH (C) genes expression using RT-PCR method. All results are expressed as the mean ± SD (n = 7). ^a^p<0.05, significant change with respect to the **Control** group; ^b^p<0.05, significant change with respect to the **Infected** group using Duncan's post hoc test.

To assess the effect of *S*. *mansoni* infection on oxidative stress profile, namely lipid peroxidation, nitrite/nitrate, and glutathione levels were determined in the liver tissues (Fig 5). Schistosomiasis induced a significant (p<0.05) elevation in the hepatic levels of LPO and NO compared with the healthy group. ZLE administration significantly (p<0.05) attenuated the elevation in LPO and NO levels in the infected groups. Furthermore, schistosomiasis was associated with a significant depletion (p<0.05) the GSH content of the hepatic tissues compared to that of the negative control group. This depletion in GSH content was minimized by post-infection medication with ZLE (Fig 5).

**Fig 6:**
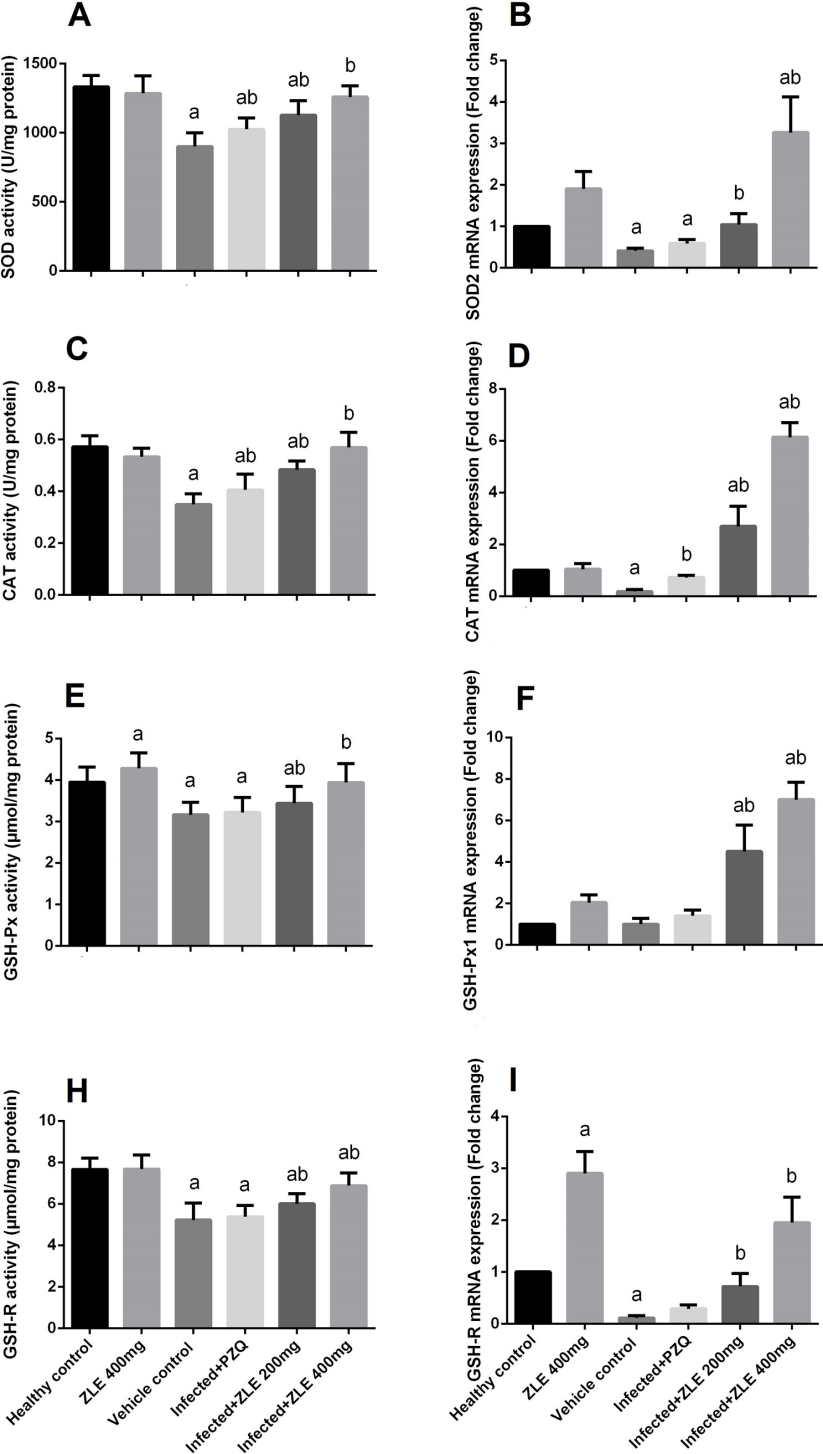
Ameliorative effects of the administration of *Ziziphus spina*-*christi* leaf extract (ZLE) on the antioxidant enzyme levels and expressions in CD-1 Swiss mice infected with *S*. *mansoni*. After 56 days, liver homogenates were prepared to assayed SOD (A) and their expression (B), CAT (C) and their expression (D), GPx (E) and their expression (F), and GR (H) and their expression (I). All biochemical results are expressed as the mean ± SD (n = 7). mRNA results (mean ± SD of three independent assays) were normalized to the GAPDH mRNA level and are shown as fold induction relative to the mRNA level in the control. ^a^p<0.05, significant change with respect to the **Control** group; ^b^p<0.05, significant change with respect to the **Infected** group using Duncan's post hoc test.

To assess how schistosomiasis induces oxidative damage in hepatic tissue, the activities of the antioxidant enzyme were analyzed, with a focus on SOD, CAT, GST, GSH-Px, and GSH-R enzyme activities. As demonstrated in Fig 6, at 8 weeks post-infection by *S*. *mansoni*, significant inhibition in the antioxidant enzyme activities (p<0.05) was observed compared with that of the controls. This inhibition was significantly (p<0.05) relieved by ZLE administration. Real-time qPCR assays further showed that the expression of SOD2, CAT, GSH-Px1, and GSH-R genes decreased in the livers of the *S*. *mansoni*-infected animals but was restored in infected groups treated with ZLE.

**Fig 7:**
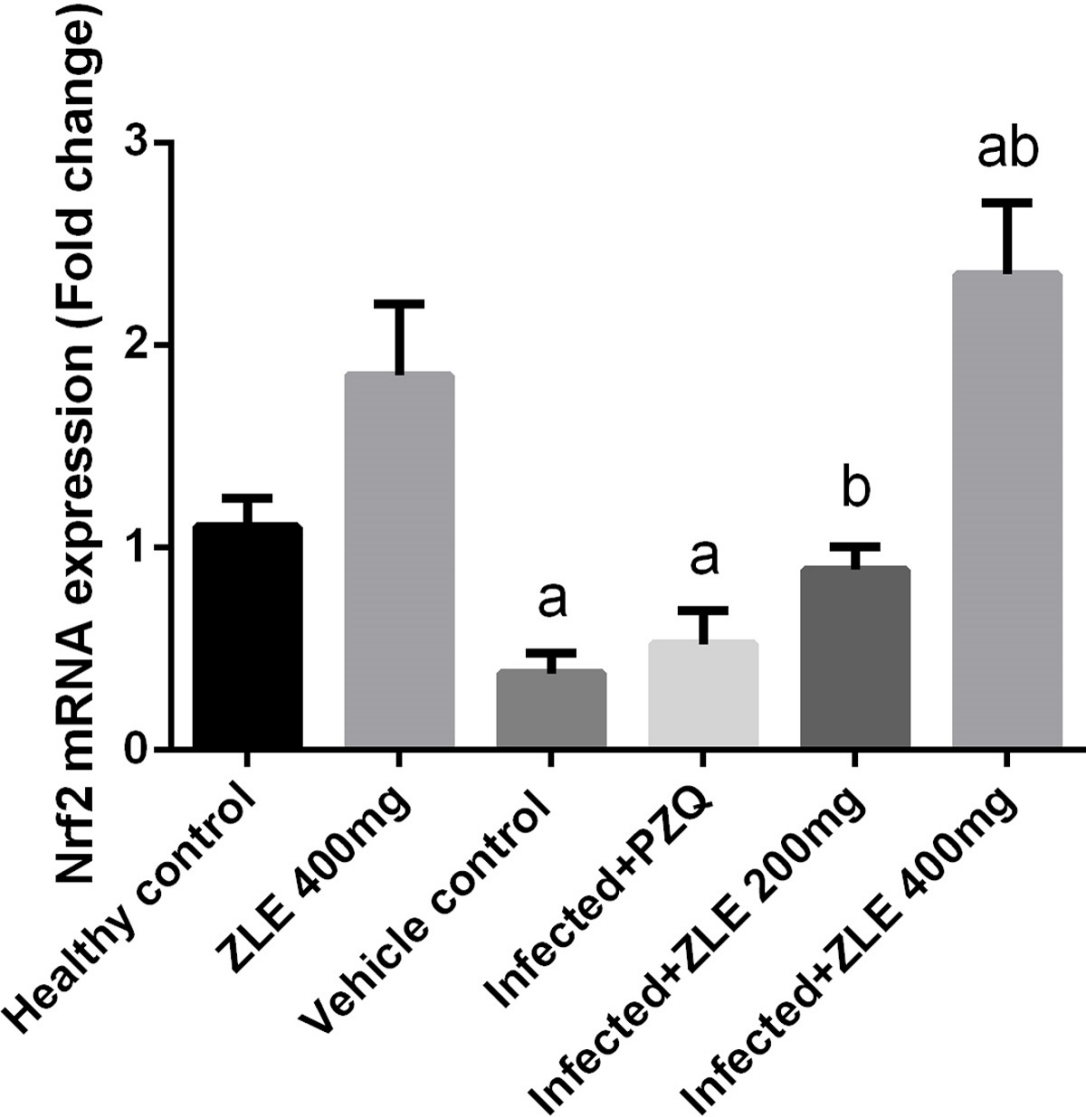
Ameliorative effects of the administration of *Ziziphus spina*-*christi* leaf extract (ZLE) on the mRNA level of Nrf2 gene in the liver of CD-1 Swiss mice infected with *S*. *mansoni*. Results (mean ± SD of three independent assays) were normalized to the GAPDH mRNA level and are shown as fold induction relative to the mRNA level in the control. ^a^p<0.05, significant change with respect to the **Control** group; ^b^p<0.05, significant change with respect to the **Infected** group using Duncan's post hoc test.

Nrf2 is a master regulator of oxidative damage via its ability to enhance the expression of hundreds of antioxidant and detoxifying genes. To confirm the antioxidant potential of ZLE in *S*. *mansoni*-induced liver damage, we determined the expression of Nrf2. The mRNA expression levels of Nrf2 in livers of mice infected with *S*. *mansoni* and treated with ZLE or PZQ are shown in Fig 7. Nrf2 expression in mice infected with *S*. *mansoni* was significantly decreased (p<0.05) and PZQ was failed to increase this expression. Interestingly, ZLE at higher dose was able to up-regulate the expression of Nrf2 in mice infected with *S*. *mansoni*.

**Fig 8:**
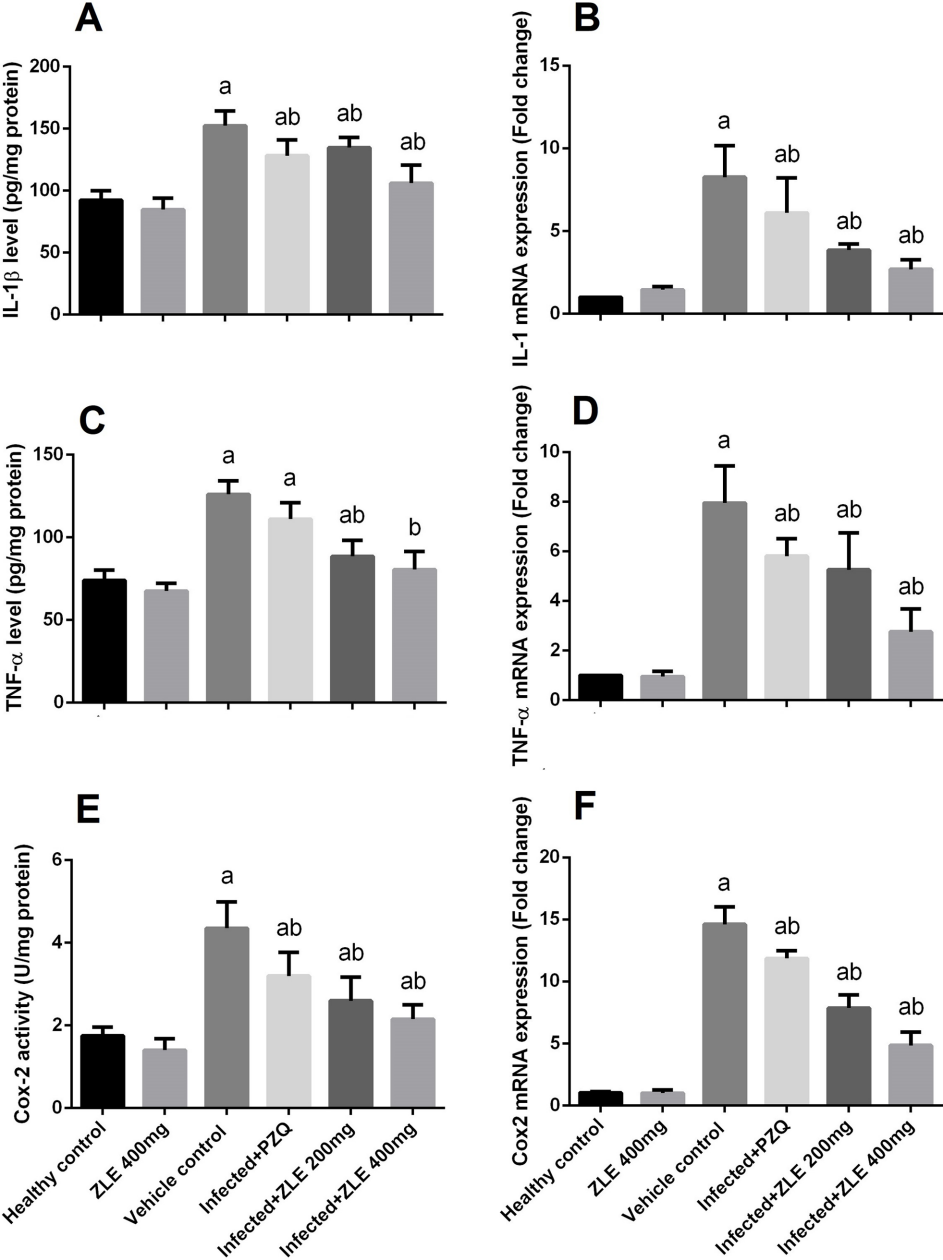
Ameliorative effects of the administration of *Ziziphus spina*-*christi* leaf extract (ZLE) on the markers of inflammation levels and mRNA expressions in CD-1 Swiss mice infected with *S*. *mansoni*. After 56 days, liver homogenates were prepared to assayed IL-1β (A) and their expression (B), TNF-α (C) and their expression (D) and Cox-2 (E) and their expression (F). All biochemical results are expressed as the mean ± SD (n = 7). mRNA results (mean ± SD of three independent assays) were normalized to the GAPDH mRNA level and are shown as fold induction relative to the mRNA level in the control. ^a^p<0.05, significant change with respect to the **Control** group; ^b^p<0.05, significant change with respect to the **Infected** group using Duncan's post hoc test.

The mRNA expression of IL-1β, TNF-α, and Cox-2 along with their levels were studied to prove the anti-inflammatory activity of ZLE (Fig 8). Although *S*. *mansoni* infection induced pronounced hepatic inflammation, a remarkable increase in the levels of IL-1β, TNF-α, and COX-2 was observed in the liver tissues of the infected mice and the mRNA expressions of IL-1β, TNF-α, and COX-2 were also markedly increased compared with the non-infected group. In contrast, the concentration of the pro-inflammatory cytokine in hepatic tissues was effectively diminished in the ZLE treatment groups, and different dosage of ZLE diminished the mRNA expression in a dose-dependent fashion, suggesting the ability of ZLE in preventing *S*. *mansoni*-induced hepatic injury.

**Fig 9:**
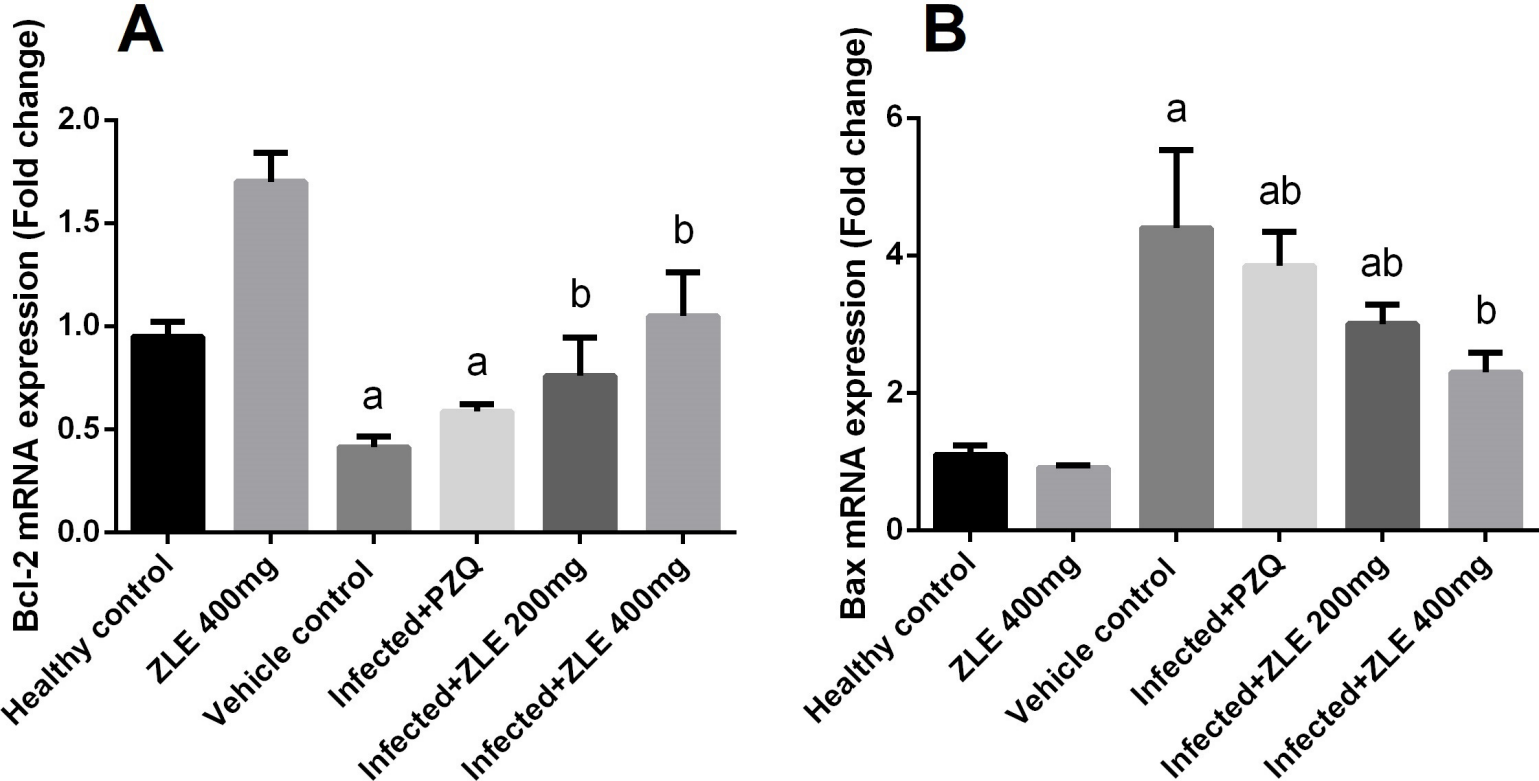
Ameliorative effects of the administration of *Ziziphus spina*-*christi* leaf extract (ZLE) on the mRNA level of apoptosis genes (Bcl-2, an anti-apoptotic gene; Bax, a pro-apoptotic gene) in the liver of CD-1 Swiss mice infected with *S*. *mansoni*. After 56 days, liver homogenates were prepared to assayed Bcl-2 (A) and Bax (B) genes expression using RT-PCR method. Results (mean ± SD of three independent assays) were normalized to the GAPDH mRNA level and are shown as fold induction relative to the mRNA level in the control. ^a^p<0.05, significant change with respect to the **Control** group; ^b^p<0.05, significant change with respect to the **Infected** group using Duncan's post hoc test.

To investigate whether the observed hepatoprotective effects of ZLE against *S*. *mansoni*-induced liver damage were related to the anti-apoptotic property of ZLE, Bcl-2 and Bax mRNA expression in the hepatic tissue were analyzed. The results showed that the mRNA expression of Bcl-2 was significantly downregulated (Fig 9), whereas that of Bax was upregulated in the *S*. *mansoni* infected mice (p<0.05). However, mice treated with ZLE showed significant upregulation of Bcl-2 and downregulation of Bax.

**Fig 10:**
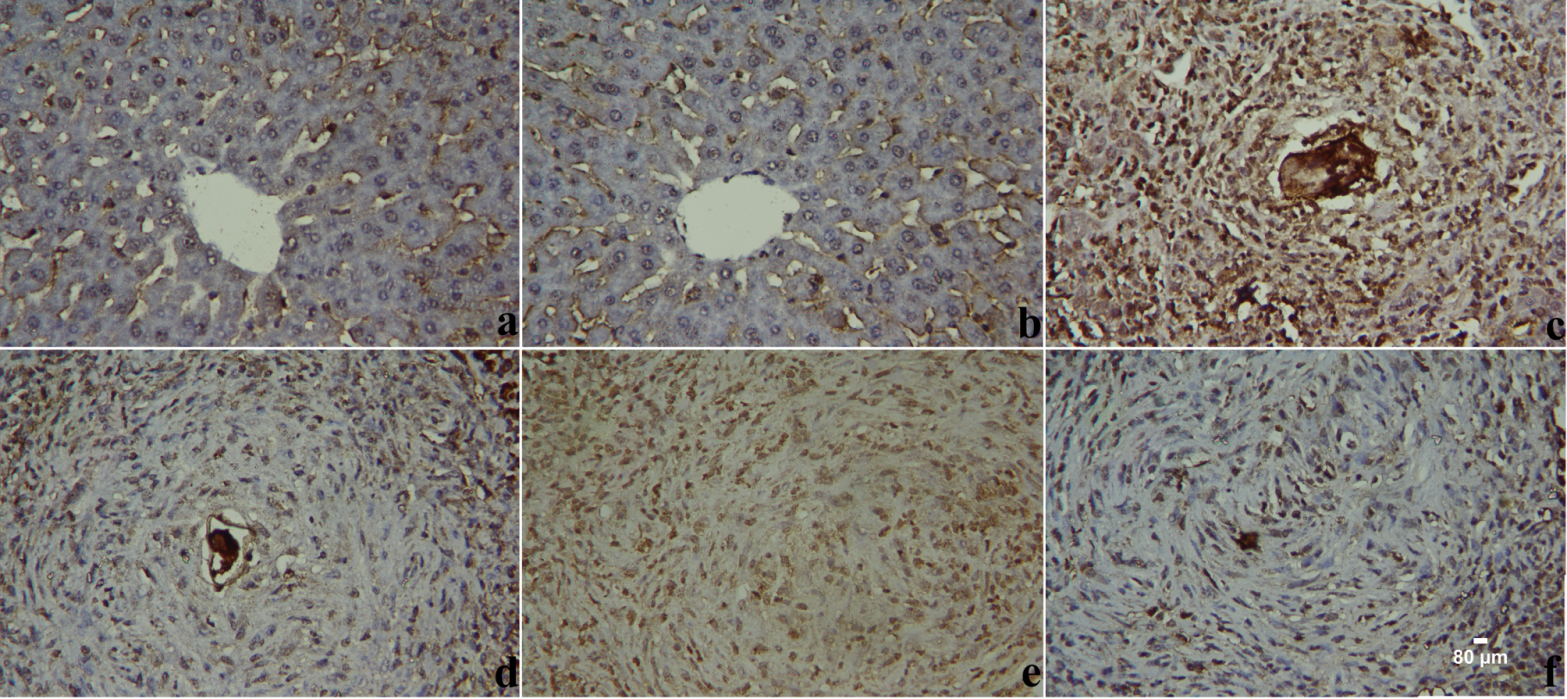
ZLE ameliorates liver apoptosis of *S*. *mansoni* infected CD-1 Swiss mice. Images representative immunohistochemistry for caspases-3 in hepatic tissue of male CD-1 Swiss mice infected with 70±5 *S*. *mansoni* cercariae for six weeks. Mice receiving saline (a), ZLE 400 mg/kg alone (b), vehicle (c), PZQ (d) and ZLE 200 (e) and 400 (f) mg/kg for two weeks. (Original magnification 400×).

In parallel with real-time PCR, immunohistochemical analysis of caspases-3 expression disclosed minimal immunoreactivity in liver tissues of the negative control group but the expression of caspases-3 was increased in the liver tissues of infected and PZQ-treated animals (Fig 10). On the other hand, ZLE administration resulted in weak to moderate caspases-3 immunoreactivity in the hepatocytes surrounding the fibrous granulomas in the hepatic tissue (S2 Table).

## Discussion

Liver fibrosis caused by *S*. *mansoni* is one of the most serious pathological changes that can induce loss of liver function and liver cancer [26]. Owing to the early, aggregate, and massive egg-laying characteristics of *S*. *mansoni*, liver fibrosis symptoms caused by *S*. *mansoni* are the most grievous among those of the prevalent schistosomiasis. Hence, identifying effective measures to prevent or even reverse liver fibrosis is critical in combating *S*. *mansoni* infection. However, safe drug intervention will not be possible until we understand the immunopathogenesis and mechanism of liver fibrosis of this disease.

Although anti-schistosomal drugs efficiently eradicate the developed worms and stop the schistosome eggs accumulation, such treatments still cannot reverse the existing liver fibrosis, particularly at chronic and complex stages of schistosomiasis. Hence, a cure of liver fibrosis produced by schistosomiasis continues to be a challenging task [4]. In the present study, *S*. *mansoni* infected mice devolved hepatic granuloma near the accumulated schistosome eggs, thereby impacting hepatocellular function. The present results demonstrating an increase in both AST and ALT indicate that chronic liver damage [27], granuloma leads to an inflammatory condition on the tissues. Granuloma size is proportional to the persistence of the egg in the lesion and the capability of the host cells to damage antigens. Granulomas are characterized by a central growth of mononuclear cells, primarily macrophages, with a surrounding rim containing fibroblasts and lymphocytes. The granulomatous reaction serves a vital function of the shielding host tissues for sequestering antigens released by eggs, and affecting pathogenesis [28]. However, in the current study, treatment with ZLE resulted in granuloma size reduction that may be due to suppression of T-cells proliferation and their cytokines which mediate granuloma formation and maturation. It has been well established that *Ziziphus* extract exerts immunosuppressive and anti-inflammatory effects in experimental studies [29]. Furthermore, ZLE has also been shown to aid restoration of normal liver cells appearance by scavenging free radicals.

In the present study, treatment of mice infected with *S*. *mansoni* with ZLE resulted in a significant decline in the number of worms and eggs along with a marginal growth in percentage of dead eggs 8 weeks post-infection. This was correlated with curing of hepatic granulomatous wounds as shown by histopathology, as well as with a decrease in the granuloma size, more granuloma constraint, more deterioration of ova, and a low number of inflammatory cells. This could be due to the anti-inflammatory and anti-oxidative properties of ZLE [30]. Further, it is likely that ZLE removes the products of oxidative reactions and aids immune-mediated devastation of worms and eggs. In addition, treatment with ZLE, in either acute or chronic infection, substantially decreased the expression of MMP-9 and TGF-β1 in the hepatic tissue. This is consistent with prior studies demonstrating that ZLE decreases the hepatic collagen content in CCl_4_ administered rats [31], exerting its anti-fibrotic properties by decreasing TGF-β and α-SMA expression and inducing *de novo* synthesis of collagen type III. TGF-β1 is a potent pro-fibrotic cytokine that encourages hepatic stellate cell stimulation, upregulates TIMPs, and downregulates MMPs [32].

Liver fibrosis in schistosomiasis is linked to the presence of eggs and host reactions. Alpha-smooth muscle actin has been associated with liver injury and fibrogenesis. Hepatic stellate cells or myofibroblasts may express α-SMA. However, following chronic liver injury, HSCs change from a quiescent form to an activated form, begin to express α-SMA, and produce the extracellular matrix (ECM). Activated HSCs are shown to exist in the boundary of egg granulomas in murine and human schistosomiasis infection [33]. Thus, in the current study, we suggested that HSCs may play a pivotal role in *S*. *mansoni-*induced liver fibrosis. HSCs secreted several cytokines, including an important cytokine in granuloma formation, TGF-β, and overexpression of TGF-β promotes HSC survival in the damaged liver [34]. Li et al. [35] suggested that TGF-β contributes not only in the inflammatory process, but also in the pathogenesis of human *schistosomal* hepatic fibrosis. Growing evidence suggests a role of TGF-β in activation of quiescent HSCs, resulting in their transdifferentiation into proliferative, fibrogenic, and contractile myofibroblasts [36]. Blockage of TGF-β signal transduction successfully inhibits the production of hepatic fibrosis [37]. In our study, TGF-β expression was observed in liver tissue and frequently observed close to granulomas. These observations suggest that HSCs as well hepatocytes play a crucial role as a source of fibrotic signals in schistosomiasis-induced liver fibrosis.

In the present study, MMP-9 expression was upregulated in hepatic tissue of infected mice, and ZLE can modulate MMP-9 expression indicating that ZLE has anti-fibrogenic activity. MMP-9 is considered to be a hallmark of fibrosis and its expression is increased by both TGF-β and TNF-α during the onset of liver fibrogenesis [8]. *S*. *mansoni* eggs stimulate the capability of HSC to rearrange the surrounding matrix. The eggs also stimulated high levels of MMP-9 expression, implying that MMP-9 digests the basement crust in the area of the eggs unaffected by its inhibitors. This reinforces the observation that myofibroblasts isolated from the granuloma tissue express MMP-9. This has also been described for other cell types [38]. In the liver, where schistosoma eggs are entrapped, MMP-9 expression might reduce the inflow of inflammatory cells. MMP-9 expression is vital for recruitment of T-cells and neutrophils in a model of post-ischemic liver illness. Moreover, MMP-9 is associated with early stage of the recruitment cascades of neutrophils, wherein inhibition of MMP-9 causes a decrease in the migration of neutrophils [39]. Previous studies also indicate that MMP-9 deficiency caused a reduction in leukocyte traffic in transwell filters, along with interrupted neutrophil migration across fibronectin [40]. MMP-9 has also been shown to have a function in macrophage migration.

Dysregulation of MMP activity frequently results in tissue impairment and functional modifications [41]. Tissue inhibitors of metalloproteinases (TIMPs) are a group of a minimum of four recognized physiological inhibitors (TIMP 1–4) capable of controlling proteolytic behaviors of MMPs in tissues where MMP-9 is expressed, TIMP-1 inhibits MMP-7, MMP-3, and MMP-1. TIMP-1 expression peaks at the fibrotic stage of *S*. *mansoni* infection in mice [32], and fibrosis reversion as a reaction to PZQ medication is linked to a drastic reduction in the levels of TIMP-1 [42]. The correlation between high levels of TIMP-1 and fibrosis has been demonstrated in numerous mammal models and in cirrhotic human liver [32]. This evidence suggests that schistosome-associated fibrosis results from extreme inhibition of collagen altering through TIMP-1 and might reproduce defects in transfer of MMP synthesizing peripheral blood mononuclear cells (PBMCs) to the liver. In addition to inhibiting MMPs, TIMP-1 has anti-apoptotic and proliferative effects on fibroblasts that might account, in part, for their profibrotic activity in schistosome infection [43].

Inflammation is a common feature in many chronic liver diseases and is closely associated with the progress of liver fibrosis [33]. It has been reported that TNF-α is an acute-response cytokine and facilitates initiation of NF-κB pathway in HSCs [44]. During the etiology of schistosome infection, the formation of multi-cellular granulomatous inflammation surrounding eggs is the classic phenomenon in the liver and intestines [28]. The inflammation initially recruits numerous inflammatory cells, such as macrophages, lymphocytes, and eosinophils. The progression of the granulomatous inflammation assists HSCs to transform from a quiescent phenotype to an activated phenotype and proliferate and migrate to the peripheral regions of egg granulomas [33]. The present study demonstrated that concentrations of pro-inflammatory cytokines IL-1β, TNF-α, Cox-2 all peaked at 8 weeks post-infection. On the basis of these findings, the increased expression of these markers might be due to the inflammatory responses caused by *S*. *mansoni* eggs. It is noteworthy that ZLE has anti-inflammatory activity. We previously found that ZFE could suppress pro-inflammatory cytokine production *via* blocking of the activation of mitogen-activated protein kinase (MAPK)/NF-κB signaling pathway [45].

*Schistosoma mansoni* infection might imbalance oxidative factors by diverse mechanisms, such as egg deposition, alterations in vascular tone, and solvable immune intermediates [46]. The presence of granuloma and immune-related cell types is probably the main reason for the rise in oxidative stress in the tissue, as the immune reaction of inflammatory cells is known to be related to the production of reactive oxygen species (ROS) and oxidative injury [8]. In accordance with this, it has been reported that an increase in oxidative markers namely, NO and LPO, represents excessive production of oxidants. The enzymatic antioxidant pathways are likely unable to compensate for the escalation in oxidant creation, as SOD, CAT, GSH-Px, and GSH-R activities were decreased, together with an apparent depletion of non-enzymatic antioxidants, such as GSH. It is likely that such results are limited to granulomatous parts of the liver, as these localized regions of inflammation are rich in oxidants, which is the principal root of cellular damage and development of hepatic fibrosis [47]. The initiation of oxidative stress because of increased production of ROS or a decrease in the antioxidant system in adult *S*. *mansoni* have been considered a desirable method for new treatment approaches [48]. In our study, ZLE administration exerted a good antioxidant activity and this due to the presence of different polyphenols in the extract. Our previous study revealed that the HPLC results showed that the extract has many antioxidant agents such as catechin, gallic acid, ellagic acid, chlorogenic acid, rutin, isoquercitrin, quercetin, and kaempferol [45].

The Nrf2 pathway is important in attenuating tissue injury and inflammation. In the present study, Nrf2 expression was downregulated in the liver tissue of *S*. *mansoni* infected mice, resulting in the reduction in the expression of antioxidant enzymes. Xu et al. found that Nrf2 deficiency results in incomplete restoration of liver damage, an increased inflammatory and pro-fibrotic reaction, and aggravated liver fibrosis [49]. Wang et al. [50] validated that Nrf2 might have an adverse effect in the pathophysiology of liver fibrosis, including in schistosomiasis-associated liver fibrosis. In the current study, ZLE treatment upregulated the mRNA level of Nrf2. Nabavi et al. [51] reported that polyphenols can suppress oxidative stress and inflammation through targeting Nrf2 and consequently activating the antioxidant response element-related cytoprotective genes. Hence, the ability of ZLE to induce Nrf2 expression might result in adequate transcriptional of different antioxidant genes, and subsequently in offsetting the redox homeostasis in schistosomiasis-induced liver fibrosis and, through this mechanism, promoting survival of hepatocytes during *S*. *mansoni* infection.

In the present study, schistosoma infection was associated with apoptosis of hepatic tissue. A previous study implied that some molecules are engaged in the apoptotic pathway in schistosomiasis and that apoptosis could be significant for the relationship between parasites and their hosts [52]. Caspases perform vital functions in the control of the apoptosis in parasites [53]. Immunomodulation is an important feature of schistosome infections, with human host immune responses switching from a T helper cell type 1 (Th1) response during the early stages of infection (3–5 weeks after infection) to a Th2 response following egg deposition and granuloma formation in host tissues [54]. Soluble egg antigens (SEA) can also induce T cell apoptosis, and this is particularly important in the formation of granulomata [55].

In the present study, high levels of NO formation in reaction to schistosome disease may be considered as one of the operators accountable for persuading oxidative stress and inflicting tissue injury [56]. Increased formation of NO is involved in peroxynitrite (ONOŌ) production. ONOŌ is a reactive nitrogen species (RNS) that potently oxidizes cellular biomolecules, causing lipid peroxidation, and impairing the role of particular enzymes. NO overproduction is positively correlated with tissue fibrosis via induction of fibrogenic cytokines and boosted collagen synthesis [57].

Reversal of schistosome-induced pathology after PZQ treatment has been formerly described [58]. In comparison to that, the findings of this study show that PZQ appears to be effective in decreasing hepatic fibrosis, as demonstrated by a clear change in the hepatic expression of α-SMA, TGF-β1, TIMP-1, and MMP-9. However, the results of ZLE treated groups suggested that ZLE administration might efficiently inhibit histological and biochemical changes associated with liver fibrosis. We hypothesize that the main mechanism for anti-fibrosis effect of ZLE is the reduced synthesis of collagens by inhibiting the expression of TGF-β1, α-SMA, TIMP-1, and MMP-9. After treatment with ZLE, the levels of inflammatory markers in the liver tissue were markedly decreased. Meanwhile, ZLE could significantly limit oxidation and reinforce the antioxidant defense system.

## Conclusions

ZLE has a toxic effect on eggs and worms of *S*. *mansoni*. Moreover, the anti-oxidant and anti-fibrotic effects of ZLE were investigated in the chronic fibrogenesis induced by *S*. *mansoni* infection. The results indicated that ZLE has antifibrotic activity by reversing *S*. *mansoni*-induced liver fibrosis through reducing hepatic expression of TGF-β1, MMP-9, and TIMP-1 and increasing antioxidative status while limiting apoptosis. Furthermore, ZLE showed good results than PZQ, and this may be due to ZLE has anti-schistosomal activity, in addition to the antioxidant, anti-inflammatory and antifibrotic effects. Whereas, PZQ has anti-schistosomal and antifibrotic activities. Thus, we propose that ZLE is an appropriate and capable therapeutic agent for the treatment of schistosomal liver fibrosis. However, further studies on the mechanisms of action of ZLE during schistosomal liver fibrosis might facilitate determination of therapeutic approaches in clinical practice.

## Supporting information

S1 Table Oligonucleotide primers used for real time–polymerase chain reaction. (DOCX)

S2 Table Effect of *Ziziphus spina-christi* leaf extract (ZLE) administration on the immunohistochemistry intensity of TGF-β, MMP-9, TIMP-1 and caspase-3 in liver of *S*. *mansoni* infected mice. (DOCX)
