# Supplementary material for: Ziziphus spina-christi leaf extract ameliorates schistosomiasis liver granuloma, fibrosis, and oxidative stress through downregulation of fibrinogenic signaling in mice
Source: PLoS One. 2018 Oct 1;13(10):e0204923. doi: 10.1371/journal.pone.0204923 (PMC6166951; doi:10.1371/journal.pone.0204923)
Supplement: S1 Table — (DOCX) [file pone.0204923.s001.docx]

**S1 Table**. Oligonucleotide primers used for real time–polymerase chain reaction

| Name | Accession number | Sense (5'---3') | Antisense (5'---3') |
| --- | --- | --- | --- |
| GAPDH | NM_017008.4 | GCATCTTCTTGTGCAGTGCC | GATGGTGATGGGTTTCCCGT |
| SOD2 | NM_001270850.1 | AGCTGCACCACAGCAAGCAC | TCCACCACCCTTAGGGCTCA |
| CAT | NM_012520.2 | TCCGGGATCTTTTTAACGCCATTG | TCGAGCACGGTAGGGACAGTTCAC |
| GSH-Px1 | NM_017006.2 | CGGTTTCCCGTGCAATCAGT | ACACCGGGGACCAAATGATG |
| GSH-R | [NM_053906.2](https://www.ncbi.nlm.nih.gov/entrez/viewer.fcgi?db=nucleotide&id=309319800) | TGCACTTCCCGGTAGGAAAC | GATCGCAACTGGGGTGAGAA |
| Nrf2 | NM_031789.2 | GGTTGCCCACATTCCCAAAC | GGCTGGGAATATCCAGGGC |
| IL-1β | NM_031512.2 | GACTTCACCATGGAACCCGT | GGAGACTGCCCATTCTCGAC |
| TNF-α | [XM_008772775.2](https://www.ncbi.nlm.nih.gov/entrez/viewer.fcgi?db=nucleotide&id=1046877819) | AGAACTCAGCGAGGACACCAA | GCTTGGTGGTTTGCTACGAC |
| Cox-2 | NM_011198.4 | AGGGCCCTACCAAGATGCTA | CCCAGGTTCAATCAGCAGGT |
| αSMA | NM_007392.3 | GGCATCCACGAAACCACCTA | TTCCTGACCACTAGAGGGGG |
| TGF-β1 | NM_011577.2 | AGGAGACGGAATACAGGGCT | CCACGTAGTAGACGATGGGC |
| Bcl-2 | NM_016993.1 | CTGGTGGACAACATCGCTCTG | GGTCTGCTGACCTCACTTGTG |
| Bax | NM_017059.2 | GGCGAATTGGCGATGAACTG | ATGGTTCTGATCAGCTCGGG |

The abbreviations of the genes; GAPDH: Glyceraldehyde-3-phosphate dehydrogenase; SOD2: Manganese-dependent superoxide dismutase (MnSOD); CAT: Catalase; GSH-Px1: Glutathione peroxidase 1; GSH-R: Glutathione reductase; Nrf2: nuclear factor erythroid 2-related factor 2; IL-1β: Interleukin 1 beta; TNF-α: Tumor necrosis factor-alpha; Cox-2: Cyclooxygenase 2; αSMA: Alpha-smooth muscle actin; TGF-β1: Transforming growth factor β1; Bcl-2: B-cell lymphoma 2; Bax:  Bcl-2-like protein 4.
