# Supplementary material for: Ziziphus spina-christi leaf extract ameliorates schistosomiasis liver granuloma, fibrosis, and oxidative stress through downregulation of fibrinogenic signaling in mice
Source: PLoS One. 2018 Oct 1;13(10):e0204923. doi: 10.1371/journal.pone.0204923 (PMC6166951; doi:10.1371/journal.pone.0204923)
Supplement: S2 Table — (DOCX) [file pone.0204923.s002.docx]

**S2 Table.** Effect of *Ziziphus spina-christi* leaf extract (ZLE) administration on the immunohistochemistry intensity of TGF-β, MMP-9, TIMP-1 and caspase-3 in liver of *S*. *mansoni* infected mice.

| Groups | TGF-β | MMP-9 | TIMP-1 | Caspase-3 |
| --- | --- | --- | --- | --- |
| Healthy control | + | ++ | + | + |
| ZLE (400 mg/kg bwt) | + | + | + | + |
| Vehicle control | ++ | ++++ | +++ | ++++ |
| Infected + PZQ (500 mg/kg bwt) | ++ | ++ | ++ | ++ |
| Infected + ZLE (200 mg/kg bwt) | + | ++ | + | ++ |
| Infected + ZLE (600 mg/kg bwt) | + | ++ | + | + |

Note: + = Weak immunoreactivity, ++ = Moderate immunoreactivity, +++ = Strong immunoreactivity, and ++++ = Very strong immunoreactivity.
